# Electrochemical Characterization of a Novel Exoelectrogenic Bacterium Strain SCS5, Isolated from a Mediator-Less Microbial Fuel Cell and Phylogenetically Related to *Aeromonas jandaei*

**DOI:** 10.1264/jsme2.ME15185

**Published:** 2016-07-09

**Authors:** Subed Chandra Dev Sharma, Cuijie Feng, Jiangwei Li, Anyi Hu, Han Wang, Dan Qin, Chang-Ping Yu

**Affiliations:** 1 Key Laboratory of Urban Pollutant Conversion, Institute of Urban Environment, Chinese Academy of SciencesXiamen 361021China; 2 University of Chinese Academy of SciencesBeijing 100049China; 3 College of Ecology and Resources Engineering, Wuyi UniversityWuyishan City 354300China; 4 Graduate Institute of Environmental Engineering, National Taiwan UniversityTaipei 106Taiwan

**Keywords:** Microbial fuel cell, exoelectrogenic bacterium, iron reduction, cyclic voltammetry

## Abstract

A facultative anaerobic bacterium, designated as strain SCS5, was isolated from the anodic biofilm of a mediator-less microbial fuel cell using acetate as the electron donor and α-FeOOH as the electron acceptor. The isolate was Gram-negative, motile, and shaped as short rods (0.9–1.3 μm in length and 0.4–0.5 μm in width). A phylogenetic analysis of the 16S rRNA, *gyrB*, and *rpoD* genes suggested that strain SCS5 belonged to the *Aeromonas* genus in the *Aeromonadaceae* family and exhibited the highest 16S rRNA gene sequence similarity (99.45%) with *Aeromonas jandaei* ATCC 49568. However, phenotypic, cellular fatty acid profile, and DNA G+C content analyses revealed that there were some distinctions between strain SCS5 and the type strain *A. jandaei* ATCC 49568. The optimum growth temperature, pH, and NaCl (%) for strain SCS5 were 35°C, 7.0, and 0.5% respectively. The DNA G+C content of strain SCS5 was 59.18%. The isolate SCS5 was capable of reducing insoluble iron oxide (α-FeOOH) and transferring electrons to extracellular material (the carbon electrode). The electrochemical activity of strain SCS5 was corroborated by cyclic voltammetry and a Raman spectroscopic analysis. The cyclic voltammogram of strain SCS5 revealed two pairs of oxidation-reduction peaks under anaerobic and aerobic conditions. In contrast, no redox pair was observed for *A. jandaei* ATCC 49568. Thus, isolated strain SCS5 is a novel exoelectrogenic bacterium phylogenetically related to *A. jandaei*, but shows distinct electrochemical activity from its close relative *A. jandaei* ATCC 49568.

Microbial fuel cells (MFCs) have been considered as a promising technology over the past two decades due to their diverse applications including renewable energy generation, wastewater treatment, desalination, and bioremediation (7, 34, 41). This device has the ability to convert the chemical energy of an organic substrate to electrical energy by culturing microorganisms in specially designed reactors equipped with an anode and cathode (4). Microorganisms in MFCs are vital agents and known as exoelectrogens because of their electron-transferring ability to the exocellular material. Extracellular electron transfer may occur through the direct attachment of bacterial cellular components (membrane or pili) (5, 29) or via redox mediators (external or self-produced) (20, 36) and/or other primary metabolites (H_2_ or formate) (45). Many exoelectrogenic bacteria have been discovered from MFCs on the basis of Fe (III) ion reduction (*Shewanella putrefaciens*) (27) and dilution to extinction (*Comamonas denitrificans*) (52). These exoelectrogenic bacteria belong to diverse generic groups, including α-*Proteobacteria* (*Rhodopseudomonas*) (53), β-*Proteobacteria* (*Rhodoferax*) (9), γ-*Proteobacteria* (*Shewanella*, *Aeromonas*, and *Citrobacter*) (11, 29, 39, 54), δ-*Proteobacteria* (*Geobacter* and *Geopsychrobacter*) (5, 22), ɛ-*Proteobacteria* (*Arcobacter*) (16), *Firmicutes* (*Clostridium*) (37), *Acidobacteria* (*Geothrix*) (6), and *Actinobacteria* (*Propionibacterium*) (51). However, a wider range of exoelectrogenic bacteria has yet to be discovered. The current densities and power produced by these isolates vary due to their physiology, mechanisms of electron transfer, and the different MFC architectures used to study them. The basic microbiological characteristics that influence the efficiency of MFCs are bacterial metabolism and electron transfer ability. The electrogenic properties and various aspects of extracellular electron transfer have been defined for some pure cultures of bacteria such as *Geobacter sulfurreducens* (5), *Escherichia coli* (36), *S. putrefaciens* (27, 29), *Rhodoferax ferrireducens* (9), *Rhodopseudomonas palustris* DX-1 (53), *Arcobacter butzleri* (16), and *Citrobacter* SX-1 (54).

The capability of exoelectrogenic bacteria to utilize various substrates varies from genus to genus and even among species. *R. palustris* DX-1 has been shown to utilize a wide variety of substrates (volatile acids, yeast extract, and thiosulfate) for power production in different metabolic modes (53). *Citrobacter* SX-1 produced electricity from citrate, acetate, glucose, sucrose, glycerol, and lactose in MFCs, with the highest current density of 205 mA m^−2^ being generated from citrate (54). *Clostridium butyricum* fermented glucose to acetate, butyrate, CO_2_, and H_2_ (37), whereas *S. putrefaciens* and *G. sulfurreducens* preferentially utilized lactate and acetate, respectively (5, 29). Although significant advances have been achieved in power production by optimizing and designing bacteria and MFC architectures, there is still a limitation to MFC technology being commercialized due to its low power output. It is important to identify new bacterial species with the potential to produce electricity under various environmental conditions and also pinpoint the microbial genetics as well as biochemical routes responsible for facilitating electron transfer. Therefore, the present study was conducted in order to isolate and identify new exoelectrogenic bacteria from MFC. An exoelectrogenic bacterium has been isolated through alpha ferric oxyhydroxide (α-FeOOH) reduction and identified as a novel strain phylogenetically related to *A. jandaei* using morphological, biochemical, and molecular analyses. This isolate has been confirmed as an exoelectrogenic bacterium on the basis of cyclic voltammetry (CV) principles.

## Materials and Methods

### Isolation

Bacterial strain SCS5 was isolated from the anodic biofilm of a mediator-less MFC fed with sodium acetate and operated in the continuous mode over a period of two years (17). An MFC containing three rectangular chambers (the anodic compartment, cathodic compartment, and internal clarifier) was constructed by Plexiglas baffles that separated the chambers without a proton exchange membrane. Holes (5 mm in diameter) were made on the baffle situated between the anode and cathode compartments in order to allow wastewater to flow through the system. Carbon cloth was used as the electrode in both the anodic and cathodic chambers. The original inoculum was anaerobically enriched activated sludge collected from a local wastewater treatment plant in Xiamen, China (sampling was approved by Xiamen Water Affairs Zhonghuan Sewage Treatment). Bacterial cells were extracted from a portion of the carbon cloth anode (2 cm^2^) by shaking with glass beads (2 mm in diameter) in a sterile serum bottle containing 20 mL 1% NaCl solution. Isolation was performed by the serial dilution of a cell suspension using the Hungate roll-tube technique (23) with medium containing (L^−1^): 0.115 g NH_4_Cl, 0.026 g KH_2_PO_4_, 0.2 g yeast extract, 0.5 g cysteine hydrochloride, 1 mg resazurin, and 20 mM sodium acetate as the electron donor, α-FeOOH (20 mM) as the electron acceptor, Wolfe’s trace mineral (10 mL), and vitamins (1 mL) (31) under anaerobic conditions. α-FeOOH was prepared as described previously (30). After a 5-d incubation at 30°C, single colonies showing a black color were selected and transferred to fresh broth medium. The roll-tube procedure was repeated several times until a pure culture was obtained.

### Morphological and growth characteristics study

A morphological study on strain SCS5 cells grown overnight in Luria–Bertani (LB) medium was performed with a Hitachi S-4800 scanning electron microscope (SEM) (Hitachi, Japan) and Hitachi H-7650 transmission electron microscope (TEM) (Hitachi, Japan). A light microscope was also used to examine Gram staining reactions and motility. In SEM, bacterial cells were first washed with 100 mM phosphate buffer solution (PBS) and then fixed with 2.5% glutaraldehyde followed by dehydration with increasing concentrations of ethanol from 30 to 100%. After dehydration, the samples were dried in a critical point dryer and then sputter-coated with gold (Au) for the SEM examination (54). In TEM, cells harvested in the exponential phase were washed with PBS, followed by their suspension and attachment to a Formvar carbon-coated grid and staining with phosphotungstic acid. The growth characteristics of strain SCS5 were assessed in LB broth media with various growth parameters. The growth temperature was determined in the range of 4 to 60°C. The pH range for growth from pH 3 to 11 was examined by adjusting the pH of LB broth media with NaOH or HCl. Tolerance to NaCl was evaluated in LB broth supplemented with various percentages of NaCl (from 0 to 8% [w/v]).

### Physiological and biochemical characterization

The physiological and biochemical characteristics of strain SCS5 were investigated using routine cultivation on LB broth or agar medium at 30°C. The isolate SCS5 was biochemically and physiologically characterized by using commercial identification kits (API 20NE and API ZYM identification systems, bioMerieux) under optimized conditions according to the manufacturer’s instructions. The tests for catalase and oxidase were performed using hydrogen peroxide (Sinopharm Chemical Reagent, Shanghai, China) and bioMerieux REF 55 635 oxidase reagent (bioMerieux), respectively, as described previously (50). Carbon-source utilization tests were performed using Biolog GN2 microplates (Biolog, Hayward, CA) containing 95 defined substrates. In this test, the inoculated bacterial cell density was adjusted to 50% transmittance at 600 nm. After a 24-h incubation, the violet color developed in each well was considered to represent a positive reaction and was categorized as a weak or strong reaction depending on the intensity. Biochemical characterization and other tests on the most closely related type strain *A. jandaei* ATCC 49568 (obtained from the Bioresource Collection and Research Center, Taiwan) were also performed under the same conditions in parallel with strain SCS5.

### Antimicrobial susceptibility

In order to evaluate the antimicrobial susceptibility of strain SCS5, the agar disc diffusion method was performed according to a previous study (13) following the guidelines described in CLSI M100-S21 (12) using 100 μL of a bacterial cell suspension containing approximately 1.0×10^8^ CFU mL^−1^ with various commercially available antimicrobial discs (Hangwei^™^, Hangzhou, China). After sterilization, approximately 15 mL Mueller-Hinton agar was distributed to each sterilized Petri plate (9 cm in diameter). Antimicrobial discs were then placed onto bacteria-inoculated plates. After being kept overnight in a refrigerator at 4°C, the plates were incubated at 30°C. The diameters of the inhibition zones were measured in millimeters following a 24-h incubation. The antimicrobial agents tested were ampicillin (10 μg), chloromycetin (30 μg), carbenicillin (100 μg), cephradin (30 μg), cefobid (30 μg), ciprofloxacin (5 μg), cefalexin (30 μg), gentamicin (10 μg), rocephin (30 μg), clindamycin (2 μg), vibramycin (30 μg), erythromycin (15 μg), kanamycin (30 μg), cefazolin (30 μg), minomycin (30 μg), metronidazole (5 μg), lincomycin (2 μg), norfloxacin (10 μg), ofloxacin (5 μg), oxacillin (1 μg), penicillin G (10 μg), polymyxin B (30 μg), piperacillin (100 μg), rifampicin (5 μg), streptomycin (10 μg), co-trimoxazole (25 μg), tetracycline (30 μg), vancomycin (30 μg), furazolidone (15 μg), and neomycin (10 μg). The antimicrobial susceptibility of type strain *A. jandaei* ACTCC 49568 was also examined with these agents under the same conditions.

### PCR amplification, sequencing, and phylogenetic analysis of 16S rRNA, gyrB, and rpoD genes

The genomic DNA of the isolate SCS5 extracted using the OMEGA DNA kit (OMEGA Bio-Tek, GA, USA) was used to amplify the sequences of the 16S rRNA, *gyrB*, and *rpoD* genes. The 16S rRNA gene was amplified by PCR using a pair of universal primers: 27F (5′-AGAGTTTGATCMTGGCTCAG-3′) and 1492R (5′-GGTTAC CTTTGTTACGACTT-3′) (37). In addition, the *gyrB* and *rpoD* gene sequences of strain SCS5 were amplified by PCR according to a previous study with slight modifications using the primers *gyrB* 3F (5′-TCCGGCGGTCTGCACGGCGT-3′)/*gyrB* 14R (5′-TTGTCCG GGTTGTACTCGTC-3′) (55) and *rpoD* 70Fs (5′-ACGACTGAC CCGGTACGCATGTA-3′)/*rpoD* 70Rs (5′-ATAGAAATAACCAG ACGTAAGTT-3′) (46), respectively. The PCR mixture contained 12.5 μL Premix Taq (TaKaRa, Dalian, China), 1 μM of each primer, approximately 0.5 μg of the DNA template, and sterile deionized water to make a total reaction volume of 25 μL. PCR amplification was performed with a GenePro Thermal Cycler (Bioer, Hercules, China) and the amplification program consisted of: 1 cycle at 94°C for 5 min, followed by 30 cycles at 94°C for 45 s, at 57°C for 30 s (for 16S rRNA gene), at 55°C for 30 s (for *gyrB* gene) or at 48°C for 20 s (for *rpoD* gene), and at 72°C for 1 min, and finally 1 cycle at 72°C for 5 min. The purified PCR products of the 16S rRNA, *gyrB*, and *rpoD* genes were sequenced and analyzed by comparing and aligning with related gene sequences available in the Ez-Taxon (10) and GenBank databases using BLAST-N and Clustal-W programs. Phylogenetic trees were constructed on the basis of the neighbor-joining method (43) using Molecular Evolutionary Genetics Analysis software (MEGA5) (47).

### Average nucleotide identity (ANI) and DNA G+C content determination

ANI was calculated as an alternative to DNA–DNA hybridization according to a previous study (42). Whole genomic DNA from strain SCS5 was extracted using the Wizard SV Genomic DNA kit (Promega, USA). Sequencing was performed using an Illumina HiSeq 2500 instrument with a paired-end library (300 bp). Genome sequences were assembled *in silico* using SOAPdenovo2 (32). An ANI analysis was performed using the software JSpecies v1.2.1 (42). The DNA G+C content of strain SCS5 was determined by calculating the total amount of G and C bases from the whole genome sequence as a percentage.

### Determination of cellular fatty acids

The cellular fatty acid profile of strain SCS5 was determined using bacterial cells grown on LB agar medium at 30°C for 24 h. Cell harvesting, the saponification of lipids, methylation of fatty acid, extraction of fatty acid methyl esters (FAMEs), and washing of extracts were performed as described in the standardized protocol of the Microbial Identification System (MIDI) (44). Extracts were analyzed and quantified using an Agilent 7890 gas chromatograph (Agilent Technologies, USA). The identification and quantification of individual FAME profiles were performed using the Microbial Identification System software package, version 3.9. The cellular fatty acid profile of the closest phylogenetic relative, *A. jandaei* ATCC 49568, was also determined in parallel with that of strain SCS5 in the present study.

### Measurement of Fe (III)-reducing activity

In order to examine Fe (III) reduction activity, 20 mL medium containing sodium acetate (20 mM) as the electron donor and insoluble α-FeOOH (20 mM) as the electron acceptor was inoculated with strain SCS5 (cell density ~1×10^9^ CFU mL^−1^) under anaerobic conditions. Fe (III) reduction was monitored spectrophotometrically by measuring HCl-extractable Fe (II) using a ferrozine assay at various intervals as previously described with slight modifications (30). An aliquot of the sample (0.1 mL) was added to a vial containing 0.9 mL of 0.5 N HCl and then incubated at room temperature for 30 min followed by centrifugation at 10,000×*g*. After centrifugation, 0.1 mL of the supernatant was added to 1 mL of ferrozine (1 g L^−1^) in 50 mM HEPES (N-2-hydroxyethylpiperazine-N′-2-ethanesulfonic acid) buffer (pH 7). Following mixing and incubating for 15 min, the amount of Fe (II) was determined by measuring the absorbance of the solution at 562 nm. Fe (II) standards were prepared from ferrous ethylenediammonium sulfate. The iron reduction experiment was also performed for the type strain *A. jandaei* ATCC 49568.

### CV analysis

The electrochemical activity of strain SCS5 was examined by CV using the three-electrode electrochemical cell with a 10-mL capacity possessing a glassy carbon electrode as the working electrode, platinum (Pt) as the counter electrode, and Ag/AgCl as the reference electrode (18). The electrodes were cleaned in ethanol and rinsed with deionized water prior to use. Approximately 3 μL of bacterial cells (cell density, approximately 3×10^10^ CFU mL^−1^) were attached to the cleaned rounded planer surface of the glassy carbon electrode (3 mm in diameter) using nafion ionomer solution (5% dispersion) as a binder. Measurements were performed in 50 mM phosphate buffer solution at room temperature and atmospheric pressure under anaerobic and aerobic conditions at a scanning rate of 10 mVs^−1^ over the potential range from −0.6 to +0.7 V (vs Ag/AgCl) with or without the sodium acetate substrate. Cyclic voltammograms of the attached bacterial cells were obtained by a CHI 630 potentiostat (CH Instruments, USA) interfaced to a personal computer with software supplied by the manufacturer.

### Confocal Raman microscopy analysis

Bacterial cell suspensions of strain SCS5 after anaerobic and aerobic cultivations were used to perform confocal Raman microscopy at room temperature. Raman spectra were obtained using the LabRAM ARAMIS confocal micro-Raman system (HORIBA Jobin Yvon, France) equipped with a Leica microscope and a charge-coupled device detector. Excitation at a wavelength of 532 nm on the sample was provided by a He-Ne laser source (48). Raman signals were collected with a 50-μm optical fiber with a resolution of 4 cm^−1^. The exposure time for spectra acquisition was 5 s to obtain high-contrast resonance spectra for c-type cytochromes.

### Evaluation of electricity generation by strain SCS5 in MFC

In order to evaluate electricity generation by isolated strain SCS5, a two-chamber MFC having carbon felt electrodes separated by a proton exchange membrane was constructed according to a previous study (18). The anode (40 cm^2^) and cathode (40 cm^2^) were connected to a resistor of 1,000 Ω. The anode solution (120 mL, pH=7.1) contained (L^−1^): 20 mM sodium acetate, 0.31 g NH_4_Cl, 5 g NaCl, 0.13 g KCl, 7.8 g NaH_2_PO_4_.2H_2_O, 17.9 g Na_2_HPO_4_.12H_2_O, 10 mL minerals, and 1 mL vitamins (31). The catholyte was composed of 50 mM phosphate buffer solution (pH=7.1) with 50 mM K_3_Fe(CN)_6_. MFC inoculated with strain SCS5 (approximately 1×10^9^ CFU) was operated in the fed-batch mode under room temperature after sparging N_2_ gas into the anodic chamber. The cell voltage produced by MFC was recorded every 10 min via a data acquisition system (UBS7660-B, ZTIC, China) connected to a personal computer. Current (I) was calculated from Ohm’s law (I=V/R, where V is the cell voltage and R is the external resistance) and current density (I/A) was calculated on the basis of the anode surface area (A).

### Nucleotide sequence accession number and strain deposition

The 16S rRNA, *gyrB*, and *rpoD* gene sequences of strain SCS5 determined in this study have been deposited in the GenBank database under the accession numbers KM091920, KP754712, and KP754713 respectively. The Whole Genome Shotgun project has been deposited at DDBJ/EMBL/GenBank under the accession number LFCS00000000. The version described in this manuscript is the first version, LFCS01000000. The isolated strain SCS5 has been deposited in the Marine Culture Collection of China (MCCC 1K 00432).

## Results

### Isolation and morphological and growth characteristics of strain SCS5

**Fig. 1:**
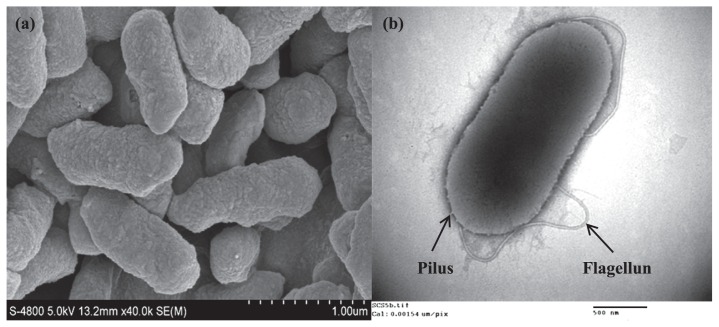
Scanning (a) and transmission (b) electron micrographs of strain SCS5.

Several bacterial cultures that formed black coloration around their colonies on the yellowish colored medium containing sodium acetate as the electron donor and α-FeOOH as the electron acceptor were isolated in a pure form (Fig. S1 in Supplemental Information [SI]). However, among these bacteria, strain SCS5 was selected for further study due to its high Fe (III) reduction activity and electrochemical properties. SEM images showed that isolated SCS5 cells were rod shaped with an approximate size of 0.9–1.3 μm in length and 0.4–0.5 μm in width (Fig. 1a). Strain SCS5 possessed a long single polar flagellum and large number of pili all over the cell that were clearly observed under TEM (Fig. 1b). The isolate SCS5 was Gram-negative and motile, as observed under a light microscope. On LB agar medium, strain SCS5 formed smooth, light gray, convex, and circular colonies with regular edges that were 1.3–1.5 mm in diameter after a 24-h incubation under aerobic conditions. The isolate SCS5 had the ability to grow under aerobic and anaerobic conditions, suggesting that strain SCS5 is a facultative anaerobic bacterium. The results for the growth parameters of strain SCS5 were shown in Table 1. The growth pH and temperature range for strain SCS5 were from 4–10 and 4–55°C, respectively. However, strain SCS5 showed good growth within a pH range of 5–9 and temperature range of 25–50°C, whereas optimum pH and temperature were 7.0 and 35°C, respectively. The isolate SCS5 had the ability to grow under 0–5% NaCl with an optimum concentration of 0.5%. The absorbance value in the case of the NaCl-free culture was very close to that of 0.5%.

### Physiological and biochemical properties

The physiological characterization results obtained for strain SCS5 by the API NE20 and APY ZYM systems were shown and compared with those of the type strain *A. jandaei* ATCC 49568 in Tables S1 and S2 in SI, respectively. Strain SCS5 had the ability to reduce nitrate to nitrite, but not to nitrogen and produced indole from tryptophane. The isolate SCS5 fermented glucose and hydrolyzed arginine, gelatin, and 4-nitrophenyl-β-D-galactopyranoside. However, esculin and urea were not hydrolyzed. In the case of assimilation reactions, strain SCS5 showed positive reactions for glucose, mannose, mannitol, N-acetyl-glucosamine, maltose, capric acid, potassium gluconate, malic acid, and tri-sodium citrate. However, strain SCS5 did not show any growth in wells containing arabinose, adipic acid, and phenyl acetic acid (Table S1 in SI). These results were similar to those obtained for *A. jandaei* ATCC 49568 in the present study. The API-ZYM test revealed that the isolate exhibited alkaline phosphatase, esterase (C4), esterase lipase (C8), lipase (C14), leucine aminopeptidase, cystine aminopeptidase, trypsine, acid phosphatase, phosphoamidase, β-galactosidase, α-glucosidase, and β-glucosaminidase activities. However, the isolate SCS5 lacked valine aminopeptidase, chymotrypsin, α-galactosidase, β-glucuronidase, β-glucosidase, α-mannosidase, and α-fucosidase activities (Table S2 in SI). In carbon utilization experiments, 95 different substrates as the sole carbon source were assessed. Out of the 95 substrates tested, strain SCS5 was able to utilize 45 compounds whereas the type strain *A. jandaei* ATCC 49568 utilized 56 compounds (Fig. S2 in SI). The key phenotypic differences of strain SCS5 from the type strain *A. jandaei* ATCC 49568 and other *Aeromonas* species were shown in Tables 1 and 2, respectively.

### Antimicrobial sensitivity

Strain SCS5 was susceptible to chloromycetin (30 μg), cephradin (30 μg), cefobid (30 μg), ciprofloxacin (5 μg), gentamicin (10 μg), rocephin (30 μg), vibramycin (30 μg), erythromycin (15 μg), kanamycin (30 μg), minomycin (30 μg), metronidazole (5 μg), norfloxacin (10 μg), ofloxacin (5 μg), polymyxin B (30 μg), piperacillin (100 μg), rifampicin (5 μg), streptomycin (10 μg), tetracycline (30 μg), vancomycin (30 μg), furazolidone (15 μg), and neomycin (10 μg), but resistant to ampicillin (10 μg), carbenicillin (100 μg), cefalexin (30 μg), clindamycin (2 μg), cefazolin (30 μg), lincomycin (2 μg), oxacillin (1 μg), penicillin G (10 μg), and co-trimoxazole (25 μg). The results of antibiotic susceptibility and resistance patterns obtained in the present study for strain SCS5 and the type strain *A. jandaei* ATCC 49568 were listed in Table S3 in SI. In many cases, the antibiotic resistivity of strain SCS5 was also similar to other *Aeromonas* species such as *A. australiensis* and *A. veronii* (3, 21).

### Phylogenetic analysis

**Fig. 2:**
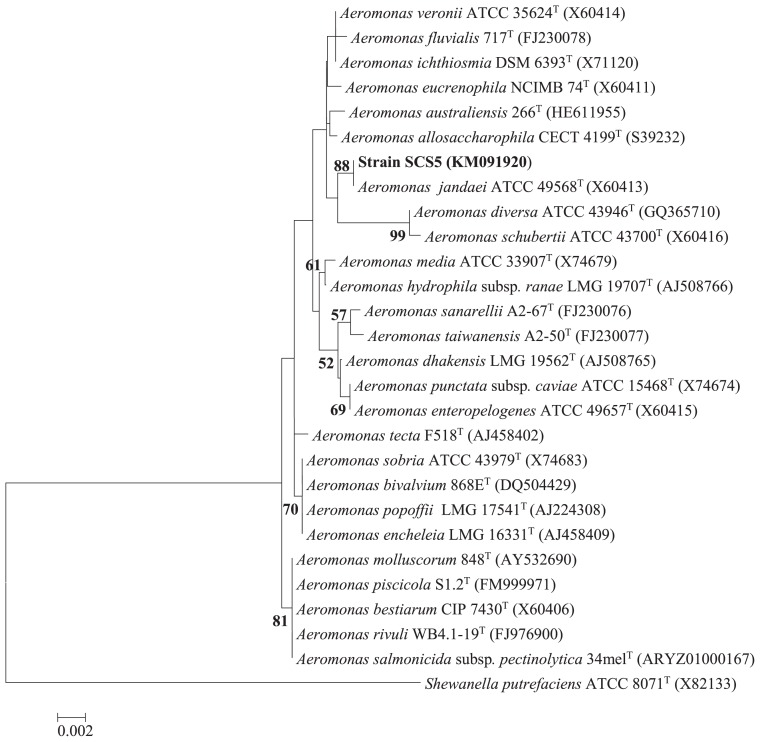
Neighbor-joining phylogenetic tree derived from 16S rRNA gene sequences showing the relationships of strain SCS5 with all other *Aeromonas* species. Numbers at nodes indicate bootstrap values >50% (expressed as percentages of 1,000 replications). *Shewanella putrefaciens* was used as the outgroup. Bar, 0.002 estimated nucleotide substitutions per site.

**Fig. 3:**
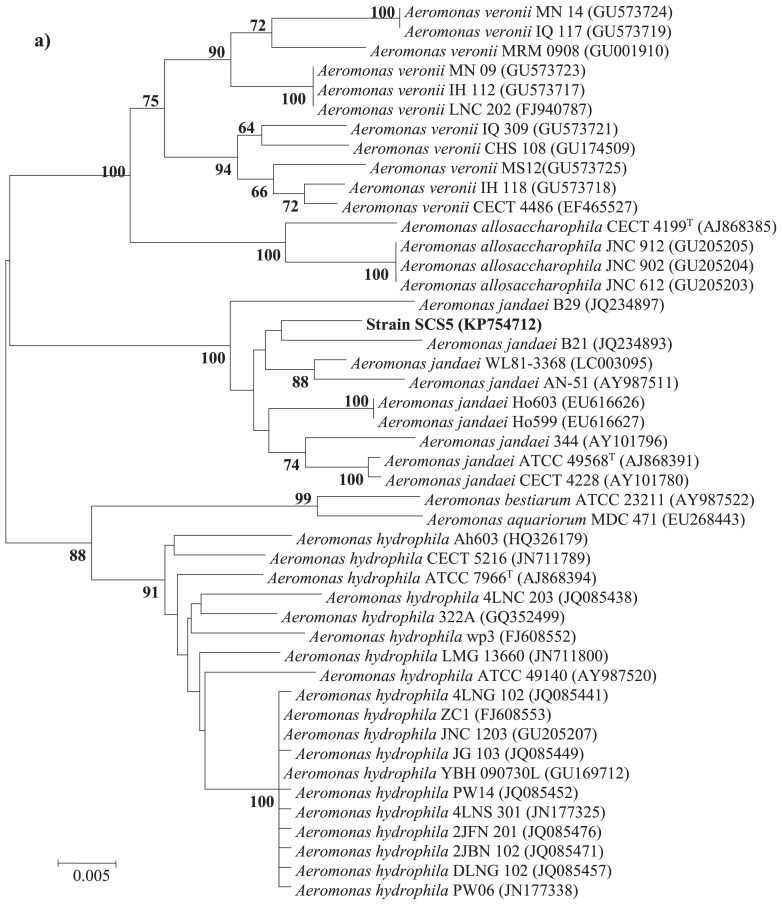

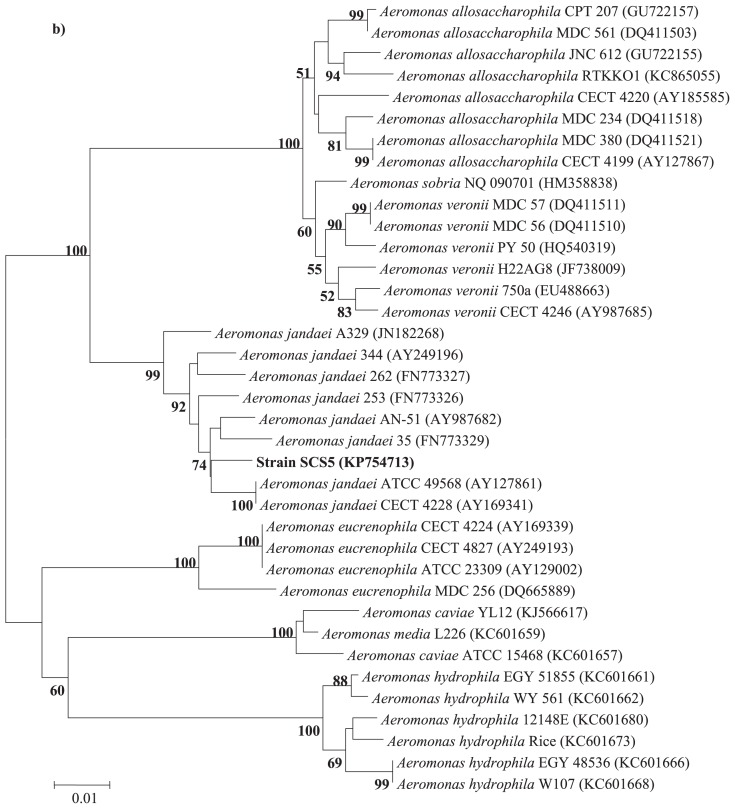
Neighbor-joining tree based on *gyrB* (a) and *rpoD* (b) gene sequences showing inter- and intra-species relationships of the genus *Aeromonas*. Numbers at nodes indicate bootstrap values >50% (expressed as percentages of 1,000 replications). Bar, 0.005 estimated nucleotide substitutions per site.

The 16S rRNA gene sequence of strain SCS5 was compared with the 16S rRNA gene sequences of *Aeromonas* members available in the Ez-Taxon and GenBank databases. The 16S rRNA gene sequence of the isolate SCS5 showed maximum sequence similarity with *A. jandaei* ATCC 49568 (99.45%), followed by *A. australiensis* 226 (99.09%), *A. veronii* ATCC 35624 (98.96%), and *A. ichthiosmia* DMS 6393 (98.96%). The phylogenetic tree constructed using the neighbor-joining method revealed that strain SCS5 and *A. jandaei* ATCC 49568 formed a tight cluster (Fig. 2). Thus, the phylogenetic analysis based on 16S rRNA genes suggests that the isolate SCS5 belongs to the genus *Aeromonas* of the family *Aeromonadaceae* in the class γ-*Proteobacteria*. The *gyrB* gene sequence of strain SCS5 showed 98% sequence similarity with several *A. jandaei* species including *A. jandaei* ATCC 49568, *A. jandaei* B21, *A. jandaei* CECT 4228, and *A. jandaei* Ho603. However, in the *gyrB* phylogenetic tree, the isolate SCS5 formed a cluster with *A. jandaei* B21 and was somewhat distantly related to *A. jandaei* ATCC 49568 (Fig. 3a). Similar to *gyrB*, the *rpoD* gene sequence exhibited 98% sequence similarity with *A. jandaei* ATCC 49568, *A. jandaei* CECT 4228, *A. jandaei* AN-51, and *A. jandaei* 344. Strain SCS5 formed a clearly distinctive branch in the phylogenetic tree, but was always clustered close to *A. jandaei* ATCC 49568 and *A. jandaei* CECT 4228 (Fig. 3b).

### ANI and DNA G+C mol % analyses

A total of 4,444,880 bp sequences were obtained from the genomic DNA of strain SCS5, providing approximately 292.4-fold coverage. Whole genome sequences from 5 closely related type strains: *A. jandaei* ATCC 49568 (accession number: CDBV01000001), *A. diversa* ATCC 43946 (CDCE01000001), *A. schubertii* ATCC 43700 (CDDB01000001), *A. allosaccharophila* CECT 4199 (CDBR01000001), and *A. australiensis* 266 (CDDH01000001), were retrieved from the GenBank database. The ANI values of strain SCS5 obtained via a BLAST analysis with respect to the above 5 strains ranged between 96.50 and 88.12% (Table 3). The ANI of the isolate SCS5 with respect to *A. jandaei* ATCC 49568 was 96.5%, which was slightly more than the threshold value of 94–96%, corresponding to the species demarcation (42) indicating a very close taxonomic relatedness between strain SCS5 and the reference strain *A. jandaei* ATCC 49568. However, the ANI values for the 4 other related strains were markedly lower than the threshold range. Thus, together with the results of the phylogenetic analysis presented above, these results suggest that strain SCS5 represents a novel strain belonging to *A. jandaei* species within the genus *Aeromonas*.

The mole percentage of the DNA G+C content of strain SCS5 determined on the basis of the whole genome sequence was 59.18% (Table 1), which was within the G+C content range of *A. jandaei* species (58.1–61.1 mol%) measured using the thermal denaturation midpoint (T_m_) (14). However, the DNA G+C content of the closest type strain *A. jandaei* ATCC 49568 calculated based on the whole genome sequence was 58.97%, indicating that the G+C content between strain SCS5 and *A. jandaei* ATCC 49568 did not markedly differ. Furthermore, the G+C content value (59.18%) obtained for strain SCS5 was within the range (57–63 mol%) reported for the genus *Aeromonas* (21).

### Cellular fatty acid composition

Cellular fatty acid contents analyzed by gas chromatography with the MIDI Microbial Identification System revealed that the most predominant cellular fatty acids of strain SCS5 (>17%) were summed feature 3 (C_16:1_*ω7c* and/or C_16:1_*ω6c*), summed feature 8 (C_18: 1_ *ω7c* and/or C_18: 1_ *ω6c*), and C_16:0_ with compositions of 33.63%, 18.4%, and 17.96% respectively (Table 4), which are similar to other *Aeromonas* species (3, 24). The fatty acid profile of the type strain *A. jandaei* ATCC 49568 examined in the present study was also shown in Table 4. Although the FAME profile was very similar, this study showed subtle differences between strain SCS5 and *A. jandaei* ATCC 49568. For example, *A. jandaei* ATCC 49568 contained C_16:0_ N alcohol (0.56%), whereas strain SCS5 did not. The fatty acid profile of strain SCS5 was consistent with most species in the *Aeromonas* genus (Table 4). The graphical data obtained from the MIDI Microbial Identification System for the isolate SCS5 and *A. jandaei* ATCC 49568 were presented in Fig. S3 in SI.

### Fe (III) reduction activity

**Fig. 4:**
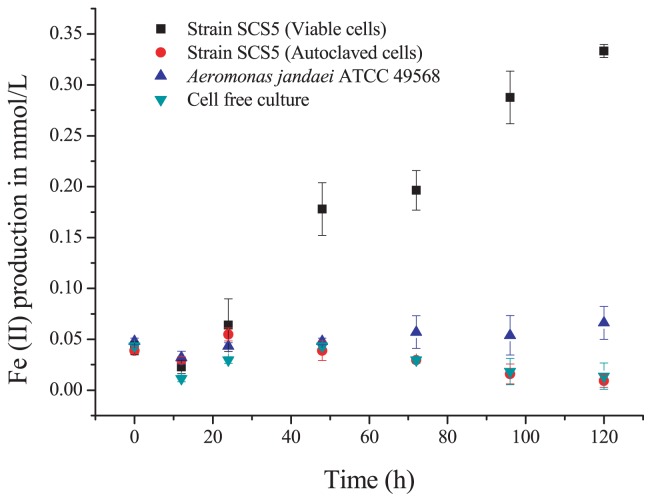
Fe (III) reduction activities of strain SCS5 and type strain *A. jandaei* ATCC 49568.

The Fe (III) reduction activity of strain SCS5 was determined by measuring the production of ferrous iron, Fe (II), in solution. The results obtained, as shown in Fig. 4, indicated that viable cells of strain SCS5 have the ability to reduce Fe (III) gradually and produce up to 0.33 mmol L^−1^ Fe (II) by 120 h. The reduction of insoluble α-FeOOH by strain SCS5 indicated that the isolate SCS5 had the ability to transfer electrons to the extracellular material. This result was consistent with those obtained for other Fe (III)-reducing bacteria including *Aeromonas* species that were electrochemically active, for example, *A. hydrophila* (39), *Aeromonas* sp. ISO2-3 (11), *Enterococcus gallinarum* (28), *S. oneidensis* MR-1 (40), *C. butyricum* (37) and *Geopsychrobacter electrodiphilus* (22). On the other hand, *A. jandaei* ATCC 49568 exhibited very low Fe (III) reduction activity. In the aspect of iron reduction activity, it is obvious that the isolate SCS5 has a distinct property from its closely related type strain *A. jandaei* ATCC 49568. The autoclaved cells and cellfree culture did not reduce α-FeOOH, which proved that the reduction of insoluble α-FeOOH exhibited by strain SCS5 was due to a physiological process, but not a chemical reaction or other exogenous products, which is consistent with previously reported findings (28).

### Characterization of electrochemical activity

**Fig. 5:**
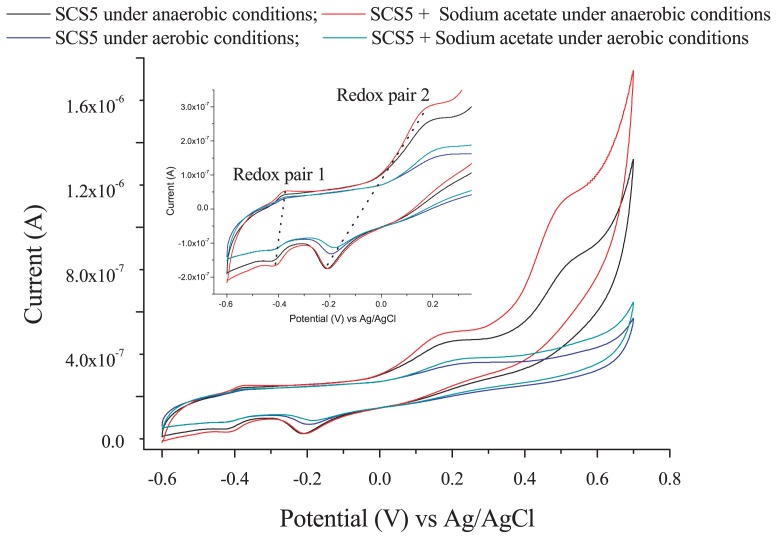
Cyclic voltammograms of strain SCS5 under anaerobic and aerobic conditions without the addition of any Fe (III) compound. The scan rate was 10 mV s^−1^. The inset shows the magnification of redox pairs 1 and 2.

**Fig. 6:**
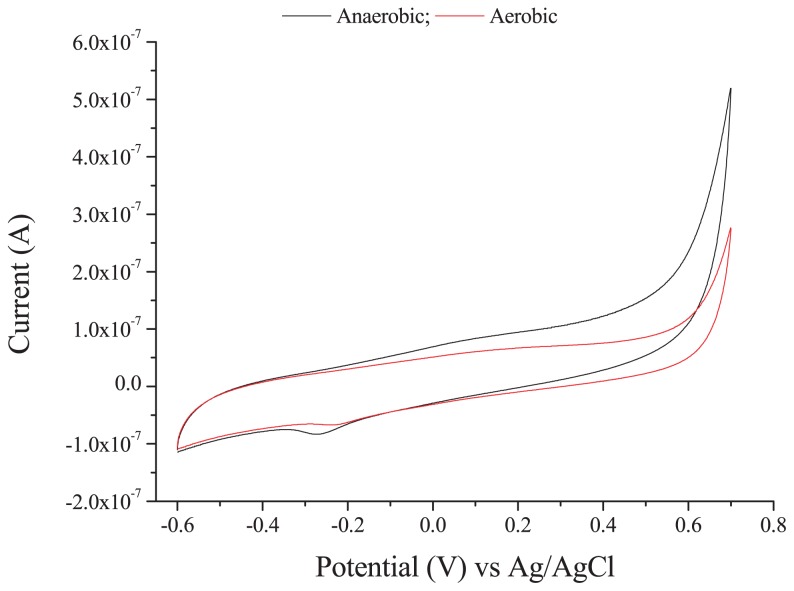
Cyclic voltammograms of *A. jandaei* ATCC 49568 under anaerobic and aerobic conditions.

In order to investigate the extracellular electron transfer capability of strain SCS5, a CV analysis was performed under anaerobic and aerobic conditions in the presence and absence of sodium acetate without the addition of any ferric compound. Two pairs of redox peaks and one individual oxidative peak appeared under anaerobic conditions, while only two pairs of redox peaks were observed under aerobic conditions (Fig. 5). The cyclic voltammograms obtained under anaerobic and aerobic conditions both showed that the oxidation and reduction peaks for the two redox pairs (redox pairs 1 and 2) were similar. The oxidative and reductive peak potentials of redox pair 1 against the saturated Ag/AgCl reference electrode were −0.380 V and −0.410 V, respectively, with an apparent midpoint potential of −0.395 V (vs Ag/AgCl). However, the apparent midpoint potential for redox pair 2 was −0.023 V (vs Ag/AgCl), at which the oxidative and reductive peak potentials were +0.166 V and −0.212 V (vs Ag/AgCl), respectively. Therefore, the standard midpoint potential for the two redox pairs (1 and 2) were approximately −0.197 V and +0.175 V against the standard hydrogen electrode (SHE, by the addition of +0.198 V), respectively. Under anaerobic conditions, there was also an additional individual oxidative peak with a potential at approximately +0.500 V (vs Ag/AgCl) or +0.698 V (vs SHE) that relatively disappeared when N_2_ gas was not purged in solution (Fig. 5). In the presence of sodium acetate, there was no marked change in voltammograms; only the height of oxidation peaks appeared more clearly than that without sodium acetate. No oxidation/reduction peak was observed in the supernatant of strain SCS5, (Fig. S4 in SI). On the other hand, *A. jandaei* ATCC 49568 did not reveal any pair of redox peaks, except for a small reductive peak (Fig. 6). These CV results demonstrate that strain SCS5 is an electrochemically active bacterium.

### Evaluation of cytochrome C content by confocal Raman microscopy

**Fig. 7:**
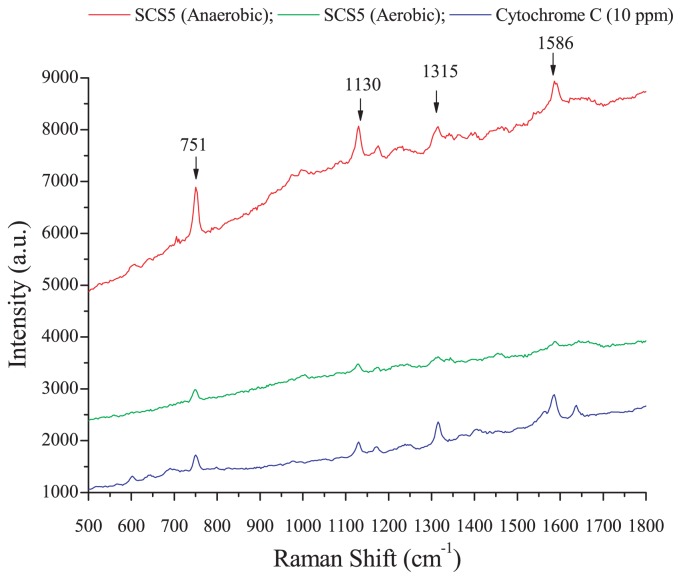
Typical Raman spectra of strain SCS5 and cytochrome C.

A Raman microscopic experiment was conducted in order to investigate whether isolate SCS5 contained cytochrome C and examine the effects of oxygen on cytochrome C content in the bacterial cell membrane. Raman spectra obtained by strain SCS5 for anaerobic and aerobic cultures and standard cytochrome C with an excitation wavelength of 532 nm at an integration time of 5 s were shown in Fig. 7. Strain SCS5 showed four strong Raman bands at 751, 1,130, 1,315, and 1,586 cm^−1^ under anaerobic and aerobic conditions, indicating the presence of cytochrome C in bacterial cellular components (Fig. 7).

### Electricity production by strain SCS5 in MFC

**Fig. 8:**
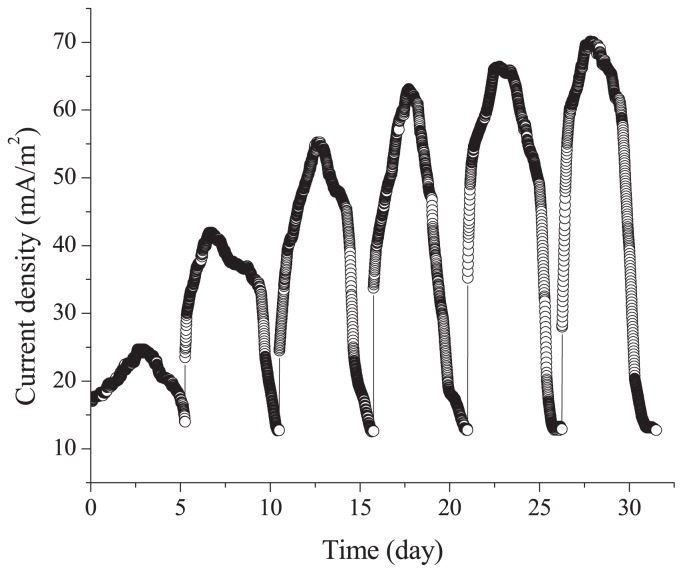
Electricity generation by strain SCS5 in MFC with sodium acetate as the substrate at 1,000 Ω.

The isolated strain SCS5 was evaluated for its ability to produce electricity using sodium acetate as the substrate in MFC at 1,000 Ω. The voltage generated by MFC inoculated with strain SCS5 gradually increased and then decreased after a period of time in each batch. However, when the MFC was refilled with fresh medium, voltage recovered rapidly and reached a higher level than that of the previous batch. Current density production increased with batch numbers from 24 mA m^−2^ to 70 mA m^−2^ and gradually reached stable states, at which MFC was operated up to six batches at 1,000 Ω (Fig. 8).

## Discussion

Comparisons of physiological and biochemical properties indicate that the characteristics of strain SCS5 are the most similar to those of members of the genus *Aeromonas*. However, there were some phenotypic characteristics that differentiated the isolate SCS5 from most *Aeromonas* species (Tables 1 and 2). Strain SCS5 was also distinct from the closest type strain *A. jandaei* ATCC 49568 (8) due to its inability to produce acid from D-sorbitol, α-cyclodextrin, N-acetyl-D-galactosamine, and D-melibiose, as well as its ability to utilize propionic acid, formic acid, β-hydroxy butyric acid, α-keto butyric acid, α-keto valeric acid, succinamic acid, and urocanic acid (Table 1). As shown in Table 2, strain SCS5 was distinguished from other *Aeromonas* relatives based on at least two or three phenotypic characteristics, as reported previously (1–3, 19, 25, 26, 38).

The phylogenetic results obtained based on the 16S rRNA gene revealed that strain SCS5 belonged to *A. jandaei* species because it formed a single cluster (Fig. 2) and had 99.45% sequence similarity with *A. jandaei* ATCC 49568. The sequence similarity value (99.45%) is greater than the threshold value of approximately 97% (typically considered for species demarcation). In order to improve the reliability of the phylogenetic analysis, two housekeeping genes (*gyrB* and *rpoD*) were sequenced and analyzed to investigate the intra- and inter-species relationship among the isolate SCS5 and other related *Aeromonas* species. In the *gyrB* and *rpoD* genes, strain SCS5 showed 98% sequence similarity with *A. jandaei* ATCC 49568, whereas in the phylogenetic tree, strain SCS5 appeared in different clustering positions. In the case of the *gyrB* gene, strain SCS5 formed a single cluster with *A. jandaei* B21, but not with *A. jandaei* ATCC 49568, which was a distant relative in the *gyrB* gene-based tree (Fig. 3a). In the case of the *rpoD* gene, strain SCS5 appeared in a distinctive branch in the tree, but as a neighbor clustering member of *A. jandaei* ATCC 49568 (Fig. 3b). Since the sequence similarity value (98%) for the *gyrB* and *rpoD* genes of strain SCS5 with *A. jandaei* species was higher than the minimal intra species similarity value of 97.7% (or within 0 to 2.3% nucleotide substitution rate) for the *gyrB* gene and of 97.4% (or within 0 to 2.6% nucleotide substitution rate) for the *rpoD* gene established in the genus *Aeromonas* (46, 55), the isolated strain SCS5 needs to be considered as a new strain phylogenetically related to *A. jandaei* species.

The CV results clearly demonstrated that strain SCS5 was an electrochemically active bacterium. The standard midpoint potential for redox pair 1 compound(s) was approximately −197 mV (vs SHE), which is almost the same as the midpoint potential (−200 mV vs SHE) of decaheme cytochrome MtrA of *S. oniedensis* MR-1 (40). The midpoint potential value −197 mV (vs SHE) was also very close to the redox potential value of approximately −190 mV (vs SHE) for membrane-associated cytochrome C of *G. sulfurreducens* (33). However, the standard midpoint potential for redox pair 2 compound(s) was approximately 175 mV (vs SHE), which is consistent with the midpoint potential (178±6 mV vs SHE) of cytochrome C-550 in the membrane of *Bacillus subtilis* (49). Therefore, cytochromes may take part in the extracellular electron transfer mechanism of strain SCS5. The midpoint potentials recorded for isolate SCS5 in the present study differed from those reported previously for *Aeromonas* species and also some other bacterial species, for example, 50 mV (vs Ag/AgCl) for *A. hydrophila* (39); 170 mV (vs Ag/AgCl) for *Aeromonas* sp. ISO2-3 (11); −200 mV (vs Ag/AgCl) for *S. putrefaciens* (29); and 200 mV (vs Ag/AgCl) for *C. butyricum* (37). The electrochemical activity exhibited by strain SCS5 was consistent with other electricity-producing *Aeromonas* species such as *A. hydrophila* and *Aeromonas* sp. ISO2-3. However, the dissimilarities of strain SCS5 to these two *Aeromonas* species were that strain SCS5 showed two redox peaks under anaerobic and aerobic conditions in the absence of Fe (III) compounds (Fig. 5), whereas *A. hydrophila* and *Aeromonas* sp. ISO2-3 only showed a redox peak under anaerobic conditions when the Fe (III) compound was in solution (11, 39). A redox peak was not found for *A. hydrophila* or *Aeromonas* sp. ISO2-3 in the presence of O_2_ or even under anaerobic conditions in the absence of Fe (III) compounds. A large number of exoelectrogenic bacteria have recently been discovered, and some of them are strictly anaerobic, such as *G. sulfurreducens*, while others are facultative such as *S. putrefaciens*. These bacteria showed redox peaks in the presence or absence of Fe (III) under anaerobic, but not aerobic conditions (29, 33, 40). It was ultimately concluded that strain SCS5 possesses potent electrochemical properties and the electrochemical reaction occurring in strain SCS5 is a quasi-reversible reaction. This exocellular electron transfer mechanism may be due to the involvement of cytochromes and/or other protein complexes.

In the present study, during the Raman microscopic experiment, four characteristic strong Raman bands at 751, 1,130, 1,315, and 1,586 cm^−1^, revealed by the cell suspension of strain SCS5, were similar to that of cytochrome C (Fig. 7), and, thus, may be ascribed to the excitation of the porphyrin ring in the heme group of cytochrome C (18, 48). Based on spectral results, it was apparent that the height of the peak intensity for the anaerobic culture was several-fold greater than that for the aerobic culture. Therefore, under anaerobic conditions, strain SCS5 contained a higher density of cytochrome C in the cellular membrane and, as such, transferred more electrons to the exocellular material than under aerobic conditions. Previous studies reported that cytochromes were localized to the outer membrane of the anaerobically grown cells of *Shewanella* and facilitated electron transfer from intact bacterial cells to electrodes (29, 35). One study also demonstrated that *c*-type cytochromes were essential for extracellular electron transfer by *G. sulfurreducens* and its electron-transferring ability depended on the content of cytochrome C (15). Thus, the results of the Raman microscopy analysis provided additional support along with the CV analysis for strain SCS5 being a potent electrochemically active bacterium. As shown in Fig. 8, it is evident that strain SCS5 is an exoelectrogen that utilizes acetate as a substrate for the production of electricity in MFC, similar to other exoelectrogens (53, 54). However, further analyses at the molecular level are needed in order to elucidate the exact mechanisms responsible for the exocellular electron transfer of strain SCS5.

## Conclusions

Based on molecular and phenotypic characterizations, we concluded that strain SCS5 isolated from MFC represents a novel strain that is phylogenetically related to *A. jandaei* in the genus *Aeromonas*. This strain possessed Fe (III) reduction activity and electrochemical properties. Strain SCS5 may potentially contribute to the generation of electricity from wastewater with the simultaneous removal of organic matter and also to the process of the iron cycle in the environment.

## Supplementary Information



## Figures and Tables

**Table 1:** Differentiation of strain SCS5 from type strains *Aeromonas jandaei* ATCC 49568^T^ and *Aeromonas jandaei* CECT 4228^T^.

Characteristics	Strain SCS5	*Aeromonas jandaei* ATCC 49568§	*Aeromonas jandaei* CECT 4228§§
Cell shape	Rod (0.4–0.5×0.9–1.3 μm)	Rod	Rod
Flagellum	Single polar	Present	Single polar
Growth:			
pH range (optimum)	4–10 (7.0)	ND	(8.5)
Temperature (°C) range (optimum)	4–55 (35)	ND	4–42
NaCl % (w/v) range (optimum)	0–5 (0.5)	No NaCl required for growth	0–3
Acid production from:			
D-Sorbitol	−	+	−
α-Cyclodextrin	−	+	ND
N-Acetyl-D-Galactosamine	−	+	ND
D-Melibiose	−	+	ND
Utilization of:			
L-Threonine	+	+	−
L-Ornithine	+	+	−
L-Alanine	+	+	−
L-Aspartate	+	+	−
L-Asparagine	+	+	−
Propionic acid	−	+	−
Formic acid	−	+	ND
β-Hydroxy butyric acid	−	+	ND
α-Keto butyric acid	−	+	ND
α-Keto valeric acid	−	+	ND
Succinamic acid	−	+	ND
Urocanic acid	−	+	ND
G+C mol %#	59.18	58.97	58.8

§ Growth characteristic parameters for *A. jandaei* ATCC 49568 were adopted from Carnehan *et al.* 1991 (8), whereas other data were obtained in our study.

§§ Data for *A. jandaei* CECT 4228 (emendation of *A. jandaei* Carnahan *et al.* 1992) were taken from Esteve *et al.* 2003 (14).

# G+C mol% based on the whole genome sequence.

(+), positive; (−), negative; ND, no data available.

**Table 2:** Key phenotypic differences in SCS5 from other *Aeromonas* species. Taxa: 1, Strain SCS5; 2, *A. australiensis* (3); 3, *A. rivuli* (19); 4, *A. fluvialis* (1); 5, *A. taiwanensis* (19); 6, *A. popofii* (25); 7, *A. sanarellii* (2); 8, *A. salmonicida* subsp. *pectinolytica* (38); 9, *A. hydrophila* subsp. *dhakensis* (26).

Characteristics	1	2	3	4	5	6	7	8	9
Esculine hydrolysis	−	−	+	−	+	−	+	−	+
Gelatine hydrolysis	+	+	+	−	+	ND	+	+	+
Indole production	+	+	−	+	+	+	+	+	+
V-P test	+	+	−	−	−	+	−	+	+
Acid production from									
Sucrose	−	+	+	+	+	−	+	+	+
Cellobiose	−	−	v	+	−	−	−	+	−
L-Arabinose	−	−	−	−	+	ND	+	+	−
Substrate utilization:									
Citrate	+	−	v	+	+	+	−	+	ND
Gluconate	+	+	−	+	+	+	+	−	+
L/D-Lactate	−	+	−	ND	ND	+	ND	−	+
Acetate	+	+	ND	ND	ND	−	ND	+	+
Urocanic acid	−	+	ND	ND	ND	ND	ND	+	+
L-Ornithine	+	−	−	−	ND	−	−	−	−
Malonate	−	−	ND	ND	ND	+	ND	ND	ND

(+), positive; (−), negative; v, variable; ND, no data available.

**Table 3:** ANI analysis between strain SCS5 (sequenced in this study) and 5 reference strains of closely related *Aeromonas* species (obtained from public repositories).

Species	Strain SCS5

	ANI (%)
*Aeromonas jandaei* ATCC 49568^T^	96.50
*Aeromonas allosaccharophila* CECT 4199^T^	88.59
*Aeromonas australiensis* 266^T^	88.12
*Aeromonas diversa* ATCC 43946^T^	81.55
*Aeromonas schubertii* ATCC 43700^T^	81.40

**Table 4:** Cellular fatty acid profiles of strain SCS5 and type strains of *Aeromonas* species identified to date. Taxa: 1, Strain SCS5; 2, *A. jandaei* ATCC 49568^T^; 3, *A. australiensis* 266^T^; 4, *A. allosaccharophila* DSM 11576^T^; 5, *A. bestiarum* ATCC 51108^T^; 6, *A. media* ATCC 33907^T^; 7, *A. caviae* ATCC 13136^T^; 8, *A. diversa* CECT 4254^T^; 9, *A. eucrenophila* ATCC 23309^T^; 10, *A. fluvialis* CECT 7401^T^; 11, *A. hydrophila* ATCC 7966^T^; 12, *A. popoffii* CIP 105493^T^; 13, *A. rivuli* CECT 7518^T^; 14, *A. salmonicida* CECT 894^T^; 15, *A. sanarellii* CECT 7402^T^; 16, *A. sobria* CIP 7433^T^; 17, *A. taiwanensis* CECT 7403^T^; 18, *A. veronii* bv. *Sobria* ATCC 9071^T^.

Fatty acid	1§	2§	3	4	5	6	7	8	9	10	11	12	13	14	15	16	17	18
C_12:0_	2.41	2.82	8.88	5.77	6.62	6.84	7.48	7.07	7.61	7.37	6.67	7.46	8.55	11.64	8.33	5.66	10.39	6.57
C_13:0_	0.28	1.21	tr	tr	tr	ND	tr	tr	tr	tr	tr	tr	tr	1.14	tr	3.6	tr	tr
C_14:0_	4.11	4.84	3.01	2.72	4.11	2.34	4.18	4.71	3.05	3.21	5.6	3.51	3.36	1.7	2.62	4.44	3.44	3.8
C_16:0_	17.96	15.46	7.55	17.34	14.96	17.36	16.25	12.06	13.51	9.66	18.28	13.68	17.14	14.51	16.93	10.75	16.09	12.28
C_17:0_	1.96	4.05	tr	tr	tr	tr	tr	tr	tr	tr	tr	tr	tr	2.14	tr	2.77	tr	tr
iso-C_13:0_	0.44	0.20	1.28	tr	tr	1.09	tr	1.21	2.11	2.66	tr	1.18	tr	ND	2.09	1.1	1.57	2.09
iso-C_15:0_	1.1	0.44	3.38	1.53	1.25	1.94	1.59	1.34	6.38	3.06	1.58	1.99	1.84	ND	2.95	2.49	2.8	3.85
iso-C_16:0_	1.04	0.25	tr	tr	tr	tr	tr	tr	tr	1.15	tr	tr	tr	tr	tr	tr	tr	tr
iso-C_15:0_ 3-OH	1.47	0.38	4.82	2.34	1.33	2.67	1.99	1.73	5.21	6.95	1.1	3.4	2.8	tr	4.48	2.68	3.48	4.4
iso-C_17:0_	2.68	0.7	1.68	1.91	tr	4.22	1.09	tr	2.83	4.08	tr	1.75	1.84	tr	3.05	1.78	1.31	3.65
C_16:1_w7c alcohol	0.17	1.39	5.8	5.18	6.52	ND	4.99	5.81	ND	2.56	5.02	6.28	tr	tr	ND	2.45	ND	5.54
C_16:0_ N alcohol	ND	0.52	1.03	1.21	1.11	ND	1.31	2.42	ND	1.94	1.67	1.62	ND	tr	ND	2.16	ND	1.11
C_17:1_w8c	2.78	6.47	1.98	tr	1.17	tr	tr	tr	tr	1.45	tr	tr	tr	2.88	tr	5.95	tr	1.42
C_17:1_ w6c	0.7	1.97	ND	ND	ND	ND	ND	ND	ND	ND	ND	ND	ND	ND	ND	ND	ND	ND
Summed feature 1*	0.28	1.3	tr	tr	tr	ND	ND	tr	ND	ND	ND	ND	ND	2.51	ND	3.16	ND	tr
Summed feature 2*	5.69	5.59	9.44	8.14	9.05	7.87	9.81	9.14	8.54	9.29	9.74	10.46	13.34	19.8	10.14	8.23	12.06	8.35
Summed feature 3*	33.63	34.66	32.37	38.11	39.2	38.42	40.67	34.28	37.94	27.3	36.55	40.55	39.04	31.31	32.93	31.19	35.5	29.9
Summed feature 8*	18.4	14.17	7.7	8.25	7.58	10.61	8.49	8.19	5.45	10.34	7.61	5.1	4.58	7.14	9.61	6.02	8.6	6.54
Summed feature 9*	2.06	0.54	3.55	2.35	1.13	4.55	1.72	1.35	5.13	3.67	1.4	2.1	1.28	tr	3.33	2.09	2.17	5.97

* Summed features contain cellular fatty acids that cannot be separated by the MIDI system. Summed feature 1 contains iso-C_15:1_ H and/or C_13:0_ 3-OH; summed feature 2 contains C_14:0_ 3-OH and/or iso-C_16:1_ I; summed feature 3 contains C_16:1_*ω7c* and/or C_16:1_*ω6c*; summed feature 8 contains C_18:1_*ω7c* and/or C_18:1_*ω6c*; summed feature 9 contains iso-C_17:1_*ω9c* and/or 10-methyl C_16:0_.

§ data obtained in our study; and other data for reference species were obtained from a previous study (3). Values are in percentages of total fatty acids; tr, trace (values <1%);

ND, no data available or not detected.
