# Supplementary material for: Electrochemical Characterization of a Novel Exoelectrogenic Bacterium Strain SCS5, Isolated from a Mediator-Less Microbial Fuel Cell and Phylogenetically Related to Aeromonas jandaei
Source: Microbes Environ. 2016 Jul 9;31(3):213–25. doi: 10.1264/jsme2.ME15185 (PMC5017797; doi:10.1264/jsme2.ME15185)
Supplement: Supplementary file 1 [file 31_213_s1.pdf]

Supplemental Materials

Electrochemical characterization of a novel exoelectrogenic bacterium strain SCS5, isolated from a mediator-less microbial fuel cell and phylogenetically related to *Aeromonas jandaei*

Subed Chandra Dev Sharma[1, 2], Cuijie Feng[1], Jiangwei Li[1], Anyi Hu[1], Han Wang[3], Dan Qin[1], Chang-Ping Yu[1, 4]*

[1] Key Laboratory of Urban Pollutant Conversion, Institute of Urban Environment, Chinese Academy of Sciences, Xiamen, 361021, China

[2] University of Chinese Academy of Sciences, Beijing, 100049, China

[3] College of Ecology and Resources Engineering, Wuyi University, Wuyishan City 354300, China

[4] Graduate Institute of Environmental Engineering, National Taiwan University, Taipei 106, Taiwan

*Corresponding author. Dr. Chang-Ping Yu, E-mail: cpyu@iue.ac.cn

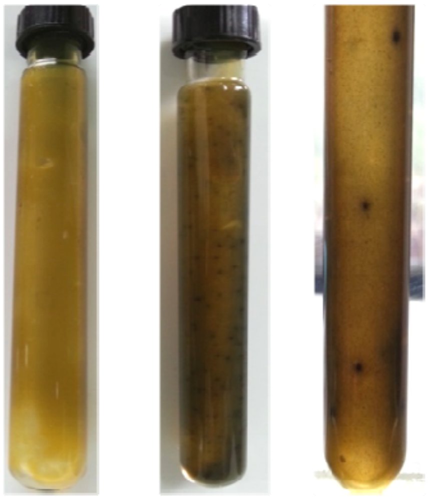

Fig S1. Photographs of pure culture isolated by roll tube method.

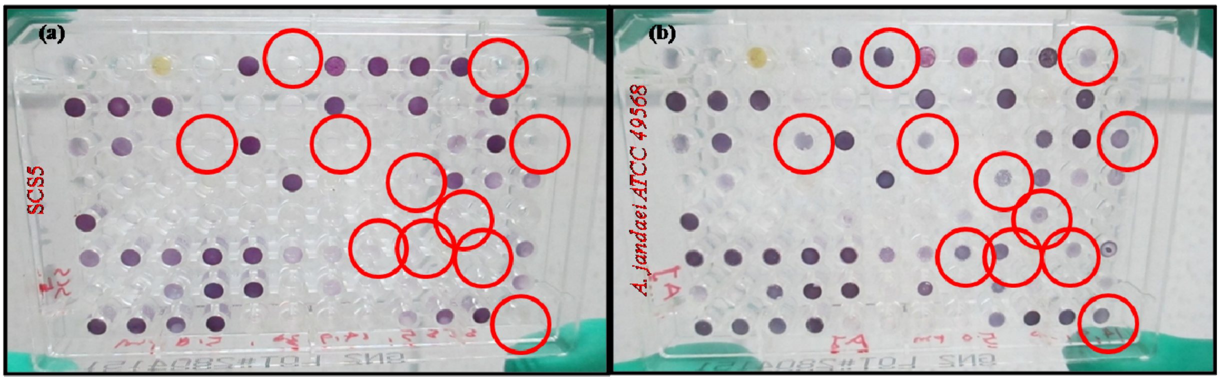

Fig S2. Utilization of substrates by strain SCS5 (a) and *Aeromonas jandaei* ATCC 49568 (b).

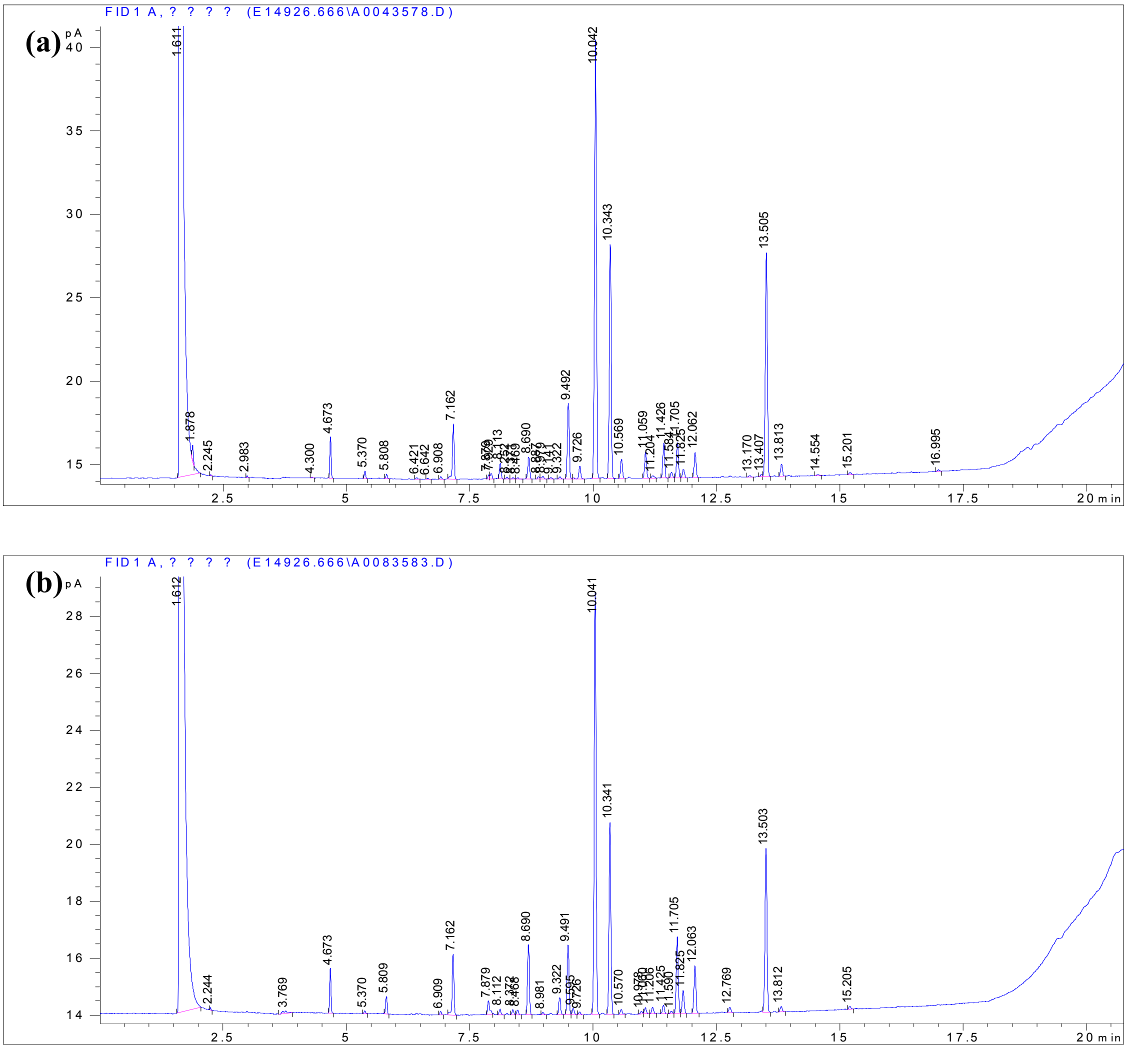

Fig S3. Graphical presentation of fatty acid profile of strain SCS5 (a) and *Aeromonas jandaei* ATCC 49568 (b).

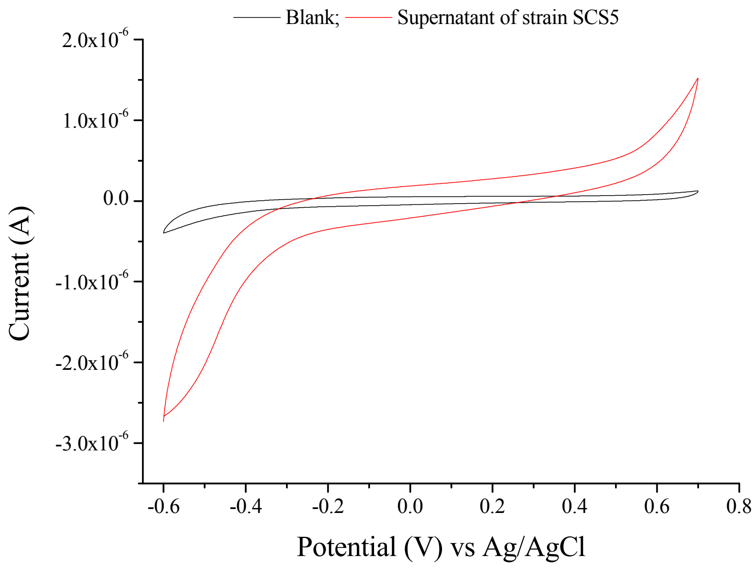

Fig S4. Cyclic voltammograms of 50 mM PBS (Blank) and supernatant of strain SCS5 under anaerobic condition.

Table S1. API 20 NE test results exhibited by strain SCS5 and *Aeromonas jandaei* ATCC 49568.

Tests	NO_3	TRP	GLU	ADH	URE	ESC	GEL	PNPG	GLU	ARA	MNE	MAN	NAG	MAL	GNT	CAP	ADI	MLT	CIT	PAC
Strain SCS5	++	++	++	++	-	-	++	++	+	-	++	++	+	++	++	+	-	++	++	-
A. jandaei ATCC 49568	++	++	++	++	-	-	++	++	+	-	++	+	++	+	++	+	-	++	++	-

(++) = Positive; (+) = Weakly Positive; (-) = Negative

Table S2. API ZYM enzymatic activity of strain SCS5 and *Aeromonas jandaei* ATCC 49568.

1. Control, **2.** Alkaline phosphatase, **3.** Esterase (C 4), **4.** Esterase lipase (C 8), **5.** Lipase (C 14), **6.** Leucine aminopeptidase, **7.** Valine aminopeptidase, **8.** Cystine aminopeptidase, **9.** Trypsine, **10.** Chymotripsin, **11.** Acid phosphatase, **12.** Phosphoamidase, **13.** α-Galactosidase, **14.** β-Galactosidase, **15.** β-Glucuronidase, **16.** α-Glucosidase, **17.** β-Glucosidase, **18.** β-Glucosaminidase, **19.** α-Mannosidase, **20.** α-Fucosidase.

**Enzymes Assayed**	**1**	**2**	**3**	**4**	**5**	**6**	**7**	**8**	**9**	**10**	**11**	**12**	**13**	**14**	**15**	**16**	**17**	**18**	**19**	**20**
Strain SCS5	-	++	++	++	++	++	-	+	++	-	++	+	-	++	-	+	-	++	-	-
A. jandaei ATCC 49568	-	++	+	++	+	++	-	-	++	-	++	+	-	++	-	-	-	++	-	-

(++) = Positive; (+) = Weakly Positive; (-) = Negative

Table S3. Antibiotic sensitivity of strain SCS5 and *Aeromonas jandaei* ATCC 49568.

Antibiotics (µg/disc)	Inhibition zone in mm	
	Strain SCS5	*A. jandaei* ATCC 49568
Ampicillin (10µg)	-	-
Chloromycetin (30µg)	45	40
Carbenicillin (100µg)	-	-
Cephradin (30µg)	20	20
Cefobid (30µg)	43	43
Ciprofloxacin (5µg)	45	42
Cefalexin (30µg)	-	15
Gentamicin (10µg)	25	24
Rocephin (30µg)	41	11
Clindamycin (2µg)	-	-
Vibramycin (30µg)	35	26
Erythromycin (15µg)	26	20
Kanamycin (30µg)	26	30
Cefazolin (30µg)	-	-
Minomycin (30µg)	33	32
Metronidazole (5µg)	32	-
Lincomycin (2µg)	-	10
Norfloxacin (10µg)	41	40
Ofloxacin (5µg)	42	43
Oxacillin (1µg)	-	-
Penicillin G (10µg)	-	-
Polymyxin B (30µg)	10	15
Piperacillin (100µg)	35	32
Rifampicin (5µg)	26	18
Streptomycin (10µg)	25	16
Co-trimoxazole (25µg)	-	26
Tetracycline (30µg)	40	34
Vancomycin (30µg)	10	8
Furozolidone (15µg)	31	27
Neomycin (10µg)	24	25
